# Geometrical Perturbation Techniques and Approximate Analysis for Eigenmode Splitting and Shifting in Electromagnetic Planar Dual-Mode Resonators

**DOI:** 10.1038/s41598-018-37787-x

**Published:** 2019-02-20

**Authors:** Adham Naji, Paul A. Warr

**Affiliations:** 1 Electrical and Electronic Engineering Department, University of Sheffield, Sheffield, S10 2TN UK; 2 Communication Systems and Networks (CSN), Faculty of Engineering, University of Bristol, Bristol, BS8 1UB UK

**Keywords:** Electrical and electronic engineering, Applied physics, Characterization and analytical techniques

## Abstract

Dual-mode electromagnetic resonators are used in numerous systems and applications in physics and engineering. They rely on degenerate-mode splitting to control the spectral properties of the system that employs them. Controlling (splitting or shifting) these eigenvalues to fully tune the frequency response, however, is a nontrivial problem that involves the use of geometrical perturbation theory as well as lossy electronic elements that enable the tuning process. In this paper we present novel geometrical techniques to control the eigenmodes of dual-mode resonators, highlighting the strong connection between the chosen geometry and performance (measured by the unloaded quality factor, *Q*_0_). Key advantages of the presented structures include electronic geometric tunability for frequency splitting and shifting, as well as the use of buried feeds to improve insertion loss and return loss performance. Field analysis is used to show how the performance is degraded by geometry itself, rather than by the tuning elements. The discussion includes derivation of approximate analytical models that highlight the sources of performance degradation in the geometry even before any tuning elements are inserted. The presented concepts are verified by measurements on perturbed microwave resonators.

## Introduction

**Figure 1:**
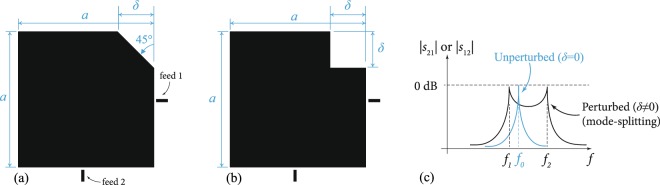
A square patch dual-mode resonator, perturbed by (**a**) a trim, or by (**b**) a square cut. This resonator has resonant wavelength *λ* ≈ 2*a* prior to perturbation. Qualitative eigenmode-splitting behavior is shown in (**c**). The scattering parameters for power transmission (*S*_21_) and reflection (*S*_11_) are used to measure the frequency response of the resonator in (**c**).

Planar dual-mode resonators are found in many systems and applications in physics and engineering, where their concept of operation relies on breaking the symmetry of an otherwise-symmetric structure to remove the degeneracy between its eigenmodes^1–10^. As a boundary value problem, the Helmholtz equation is solved over a symmetric domain that is defined by a conductive layer (e.g. square, circle, ring) on a dielectric substrate, sitting above a ground plane. The domain is then perturbed to enforce eigenmode splitting and frequency control^1^, as shown for example in Fig. 1. As the perturbation grows in size, the mode splitting increases. This process is typically done with electronic control, which means that tuning elements, such as switches or variable capacitors (varactors), are inserted to control the perturbation.

However, it is known that the addition of tuning elements to the resonator structure will typically degrade its performance (e.g. the unloaded quality factor, *Q*_0_)^4^. This degradation is mainly attributed to two sources: (1) losses due to the added active components and their associated circuitry; and (2) the fundamental loss due to geometric alterations in the resonator structure. The latter cause can be quite serious and can degrade performance simply because the chosen geometrical shape is not optimised enough to keep acceptable performance. Therefore, the way we choose to manipulate the shape or structure of the resonator to prepare it to host tuning elements may influence the performance degradation curves greatly even before actually inserting tuning elements. The performance achieved solely by geometric alterations forms an upper limit on the achievable performance when active elements are inserted^4^. Active elements (e.g. switches and varactors) will later add further losses to the fundamental (geometric) loss due to their internal losses, bias circuitry layout and wires, which can couple energy from the signal’s path, typically increasing insertion loss and causing some mismatch. This can be observed by a twofold reduction in *Q*_0_, first due to the geometrical effect and then due to the active element losses (see^4^ for an example).

This paper focused on the geometrical level, presenting and analysing novel geometrical techniques to enable eigenmode splitting and shifting (to shift centre frequency) in dual-mode resonators. Given that exact analytical solutions are not possible for the complex structures under discussion, approximate field analysis using perturbation theory is presented. Since we are not concerned here with the performance of the tuning elements themselves, these elements are assumed to be on/off switches and are replaced during modelling and measurements by ideal conductive contacts in the geometry. Note that the term *planar* here implies no field variations along substrate depth (along *z*, say), which is much smaller a dimension compared to the resonant wavelength. The latter is in the order of the planar surface dimensions (in the *xy* plane) of the resonator. Dominant modes in such structures are assumed to be ${{\rm{TM}}}_{mn}^{z}$ (transverse magnetic) type modes. Thus, a planar resonator is effectively two-dimensional (2D). For planar view clarity, the top conductor that defines the shape of the resonator is shown in all figures.

We begin by discussing novel geometrical techniques and then use perturbation theory to derive the approximate performance degradation analytically.

## Geometrical perturbation techniques for eigenmode manipulation in dual-mode resonators

**Figure 2:**
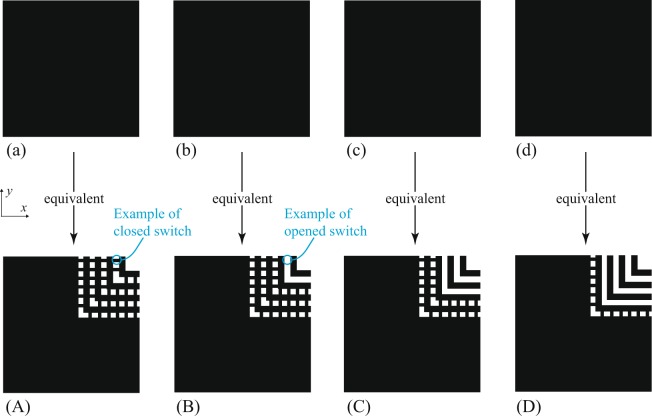
An example of a basic geometric method to achieve stepped corner perturbation inside a dual-mode square resonator. Feeds are assumed to be aligned with the *x* and *y* axes, but are not shown. The idealized switches here are small conductive strips that are switched on/off by being present/absent. As the perturbation gets larger, the eigenmode splitting is increased. Cases (**a**–**d**) are equivalent to configurations (**A**–**D**).

**Figure 3:**
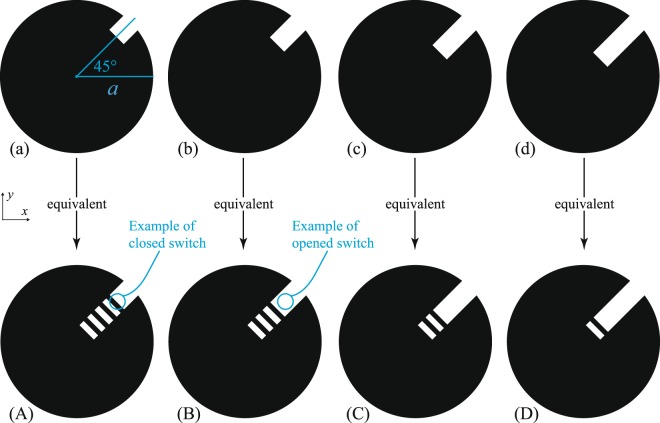
An example of a basic geometric method to achieve stepped 45°-titled perturbation inside a dual-mode disk resonator. Feeds are assumed to be aligned with the *x* and *y* axes, but are not shown. The idealized switches here are small conductive strips that are switched on/off by being present/absent. As the perturbation gets larger, the eigenmode splitting is increased. Cases (**a**–**d**) are equivalent to configurations (**A**–**D**).

A typical dual-mode resonator is the square or the circular resonator. Their inherent symmetry supports two degenerate eigenfunctions, which represent the two resonant modes of the same unperturbed frequency *f*_0_. These two modes remain orthogonal until we break the resonator’s symmetry to introduce a coupling between the two modes, elevating the resonance to second order (an example is shown in Fig. 1). This results in the characteristic mode splitting usually seen in dual-mode resonators (*f*_1_ ≠ *f*_2_), which can be controlled by changing the size of this perturbation^1^. Figures 2 and 3 show a novel geometric method on how this can be achieved for square and circular resonators.

Here, the coupling strength is changed by changing the size of the perturbation cut, which may be achieved electronically by placing rows of controlled switches to link/unlink other conductive features. The resultant apertures in the conductive face of the resonator are small relative to the resonant wavelength, but, collectively, will inevitably introduce some losses^4^ in the unloaded quality factor (*Q*_0_), which we estimate in the next section.

**Figure 4:**
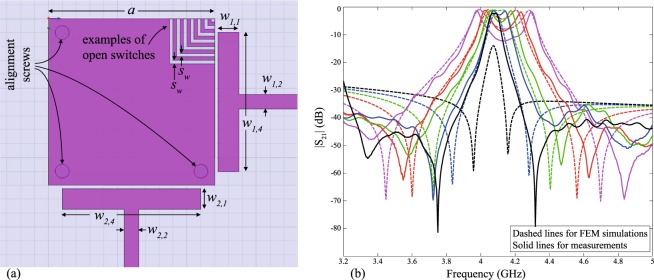
(**a**) A stripline resonator design, with air-floating feeds. This design was realized with *a* = 24 mm, sandwiched between two substrates of *ε*_*r*_ = 2.2, each substrate with *h* ≈ 3.175 mm, *w*_1,1_ = *w*_2,1_ = 3 mm, *w*_1,2_ = *w*_2,2_ = 2.1 mm, *w*_1,4_ = *w*_2,4_ = 20 mm, *s*_*w*_ = 0.5 mm, feed gap of 0.5 mm and 0.2 mm idealized switch widths. (**b**) Results of the FEM-simulation compared to measurements, for the design in (**a**), tuned in 4 steps.

**Figure 5:**
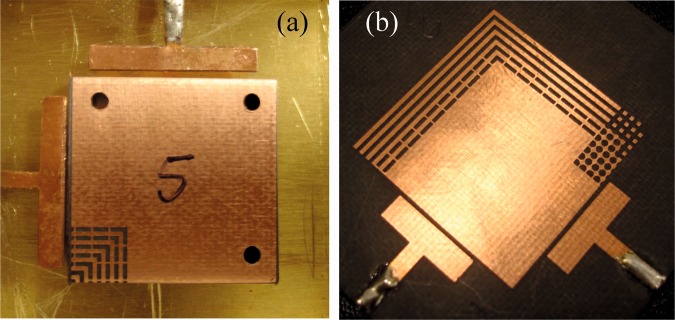
Implementation photographs of (**a**) the mode-splitting method of Fig. 2 and (**b**) the mode-splitting and frequency-shifting structure of Fig. 6. These structures were realized in stripline technology. Top substrates not shown.

The split eignevalues (*f*_1_, *f*_2_ in Fig. 1) will deviate around the original unperturbed eigenvalue (frequency *f*_0_). The deviation in frequency is governed by the cut size and shape, whereas the original value of the center frequency (*f*_0_) itself is governed by the main resonant feature (e.g. edge length or radius) in the resonator, which controls the mode under consideration. Typically, we operate the resonators at their dominant modes (e.g. ${{\rm{TM}}}_{10}^{z}$, ${{\rm{TM}}}_{01}^{z}$ in square case, and ${{\rm{TM}}}_{11}^{z}$ in circular case). This is given in terms of the side length (*a*) in the square resonator and in terms of the radius (*a*) of the circular (disk) resonators as *f*_0_ ≈ *v*/(2*a*) and *f*_0_ ≈ 1.841*v*/(2*πa*), respectively, where *v* is the velocity of light propagation in the substrate^5–7,11^. Therefore, the main resonant dimension of a resonator needs to be geometrically altered to shift the eignevalues, while the changing perturbation (cut) size controls the strength of mode splitting between them. Figures 4 and 5(a) show an implementation example of corner tuning using the method of Fig. 2. The measured performance agrees with Finite-Element Method (FEM) numerical simulations (carried out using Ansys HFSS^12^).

**Figure 6:**
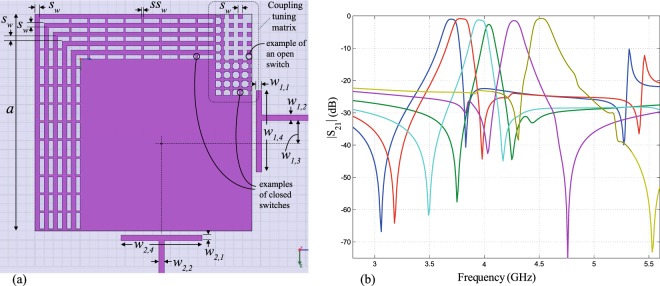
(**a**) Discussed example of a geometric method that tunes mode-splitting and center frequency. This design corresponds to Fig. 5(b). An example of frequency tuning in 6 steps (measured) is shown in (**b**).

One approach to tune the center frequency is to change the dimensions (*a* × *a*) of the square by adding/removing conductive pieces, with a similar action of switch rows. An example is shown in Fig. 6. Note that in Fig. 6, the corner perturbation is chosen to be in the form of an advanced ‘matrix’ of small conductive squares that are linked by on/off switches to achieve arbitrary cut shapes. The authors envisage this to be a form with potential application when switch technology matures enough in the future as to allow high-quality, low-cost, miniaturized switches to conveniently populate such complex structure with convenient driving circuitry (e.g. optical triggering). Even more complex geometric solutions may then evolve from Fig. 6 to enhance its features, at the expense of heavy use of miniature switches.

**Figure 7:**
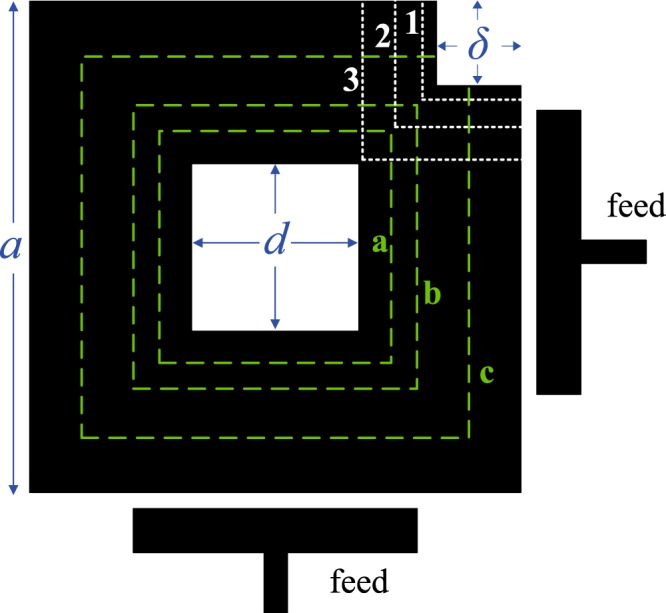
Illustration of the internal-aperture frequency-tuning concept, alongside mode-splitting tuning, applied to the square resonator. Here, *δ* controls the mode-splitting value (e.g. by advancing through positions 1, 2, 3), whereas *d* controls the center frequency value (e.g. by advancing through positions **a**–**c**).

To keep the structures feasible and minimize the degradation due to impedance mismatch and mode conversion, however, we can improve on structures like those in Fig. 6 by finding geometric solutions that keep the resonator’s main dimensions symmetric with respect to its center and its fixed feeds. One such solution is to make an aperture in the middle of the resonator, converting its shape into annular. By controlling the aperture size, we may now control *f*_0_ without changing the outer contours of the resonator or requiring the feeds to re-adjust. This concept is illustrated in Fig. 7. The resonant wavelength now is controlled by the mean perimeter inside the loop shape; the larger the aperture, the larger *λ*, and the lower the center frequency *f*_0_ = *v*/*λ*.

**Figure 8:**
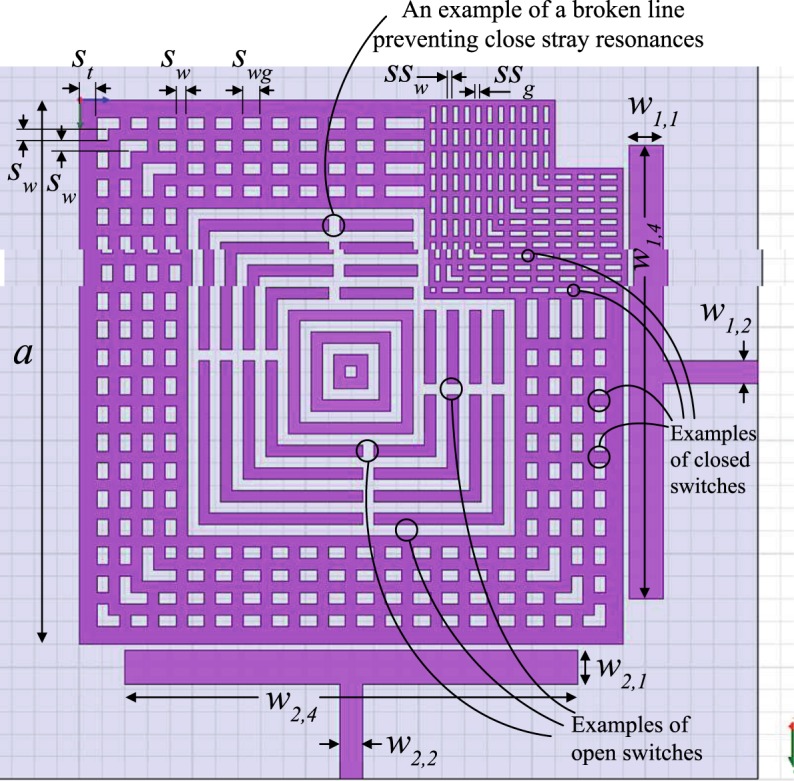
A square resonator design that applies the internal-aperture frequency-tuning concept, alongside mode-splitting.

**Figure 9:**
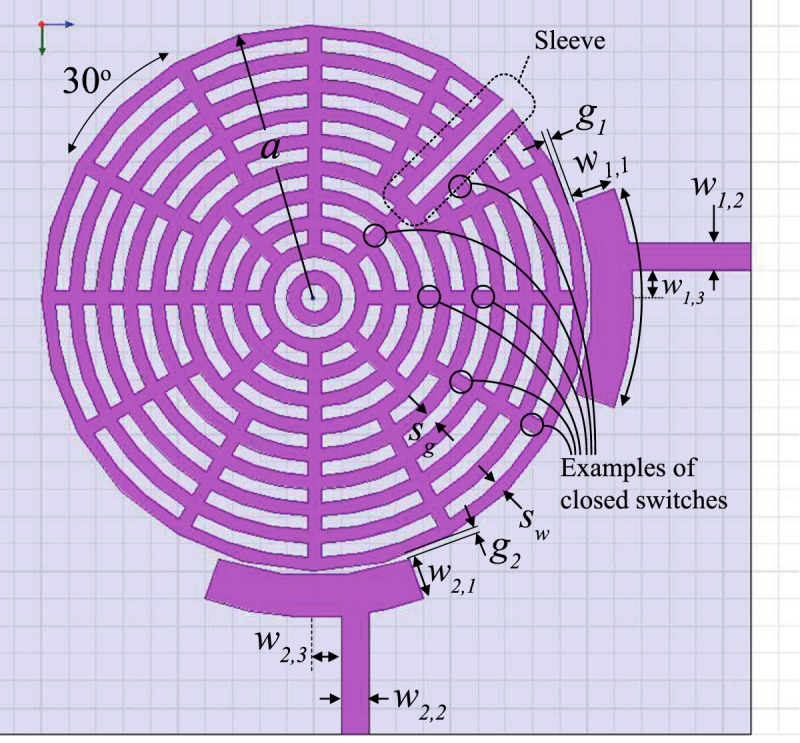
An annual resonator design that applies the internal-aperture frequency-tuning concept, alongside mode-splitting. The indicated ‘sleeve’ protects the coupling tuning switch row from the progressing of features related to frequency tuning through the internal aperture. This stripline design used a substrate with *ε*_*r*_ = 2.2, *a* = 20 mm, sandwiched between two substrates, each substrate with *h* ≈ 3.175 mm, *w*_1,1_ = *w*_2,1_ = 3 mm, *w*_1,2_ = *w*_2,2_ = 2 mm, *α* = 40°, *w*_1,3_ = *w*_2,3_ = 2 mm, *g*_1_ = *g*_2_ = 0.4 mm, *s*_*w*_ = 1 mm and *s*_*g*_ = 1 mm.

**Figure 10:**
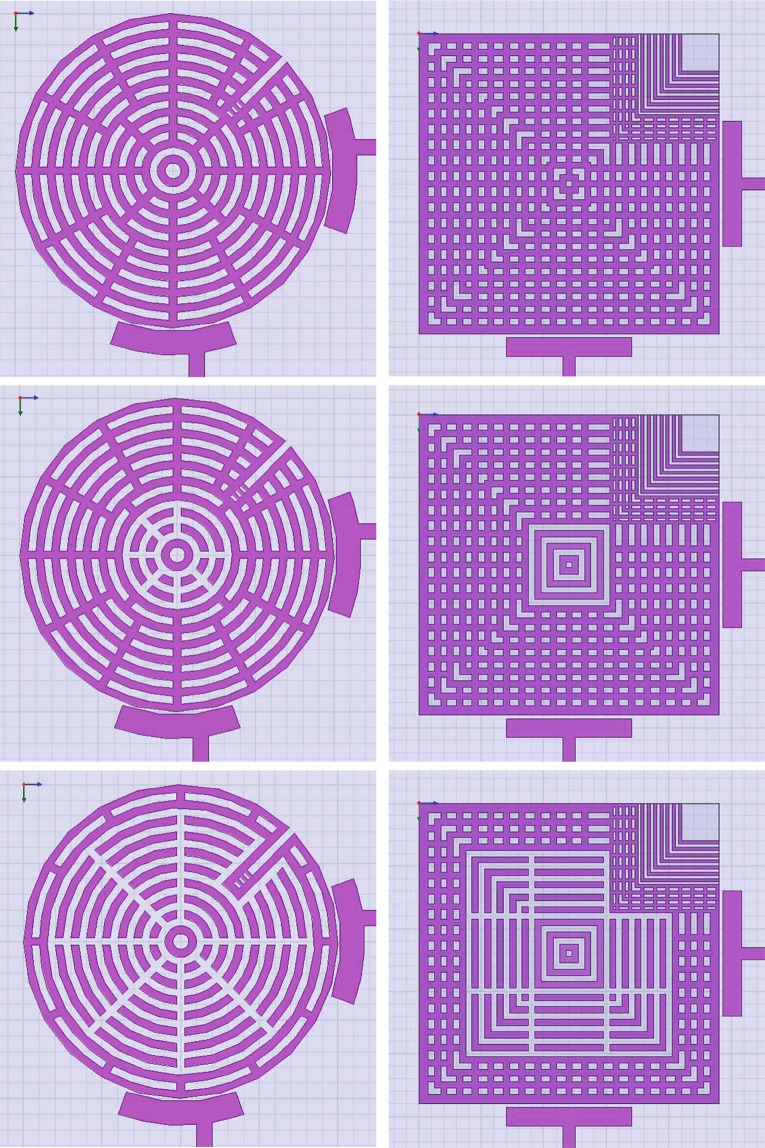
Indicative steps of frequency-tuning to illustrate the operation concept in Figs 8 and 9. As the internal gap grows, loops of conductive strips are broken into smaller segments to push their stray resonances away from the band of interest.

**Figure 11:**
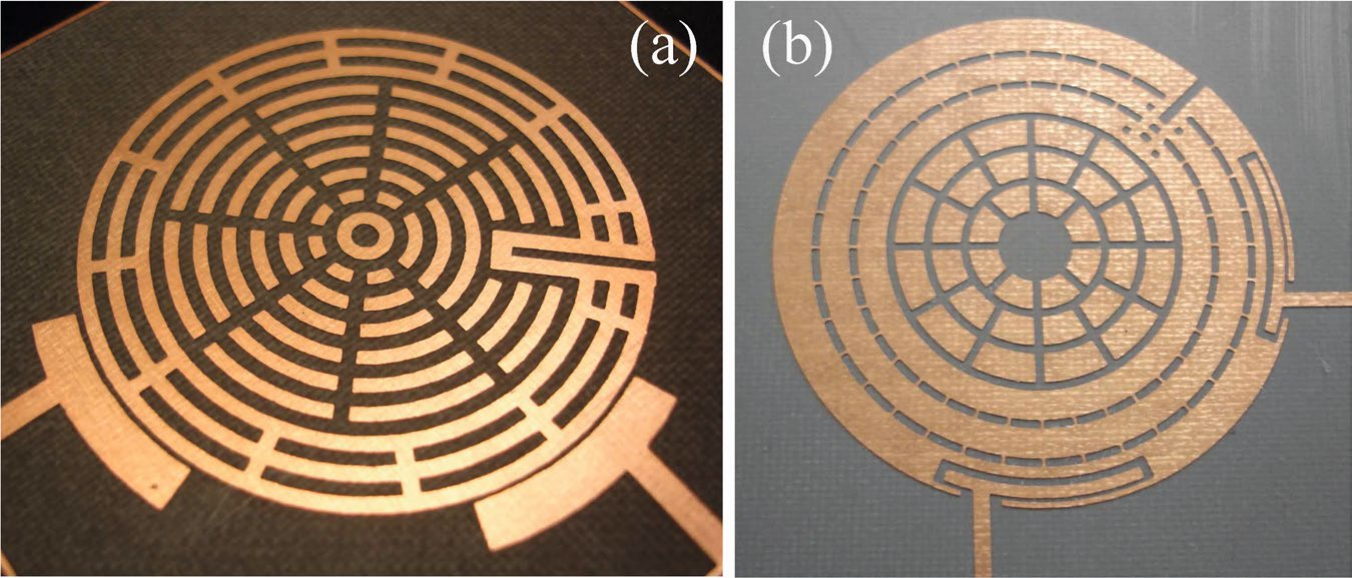
Implementation photographs of: (**a**) the concept in Fig. 9, and (b) the same concept but with pocketed feeds, as in Fig. 13. These structures are capable of both center frequency and mode-slitting tuning.

Figures 8–10 show practical square and annular shapes that apply this concept. Although the annular shape’s analysis is more involved as it contains Bessel functions, it is generally preferred in practice for its higher *Q*_0_ value, due to its smoother shape, which releases less energy to higher modes and/or radiation. The annular shape is chosen here for implementation, as shown in Fig. 11.

**Figure 12:**
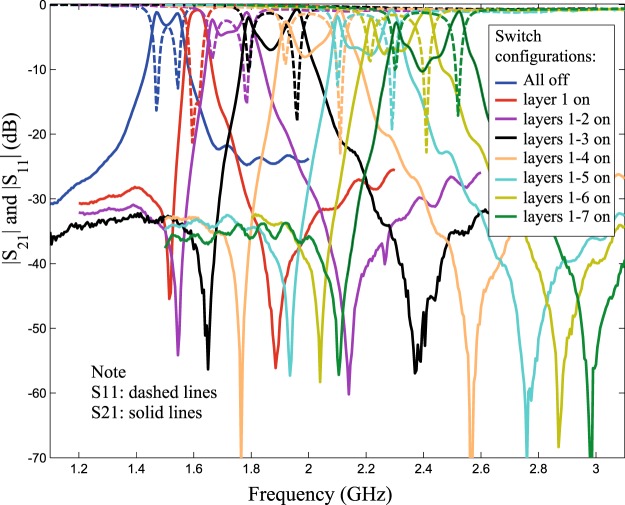
Measured results for frequency tuning steps of the structure in Fig. 9. Note that the first layer is the outermost layer.

The performance of the structure in Fig. 9 is shown in Fig. 12. Note that coupling (splitting) values will typically change when the center frequency is changed, but one can always re-tune them by adjusting the coupling-tuning switches in the sleeve, as to retain the apparent bandwidth unchanged (within the resolution of one tuning step), if desired.

**Figure 13:**
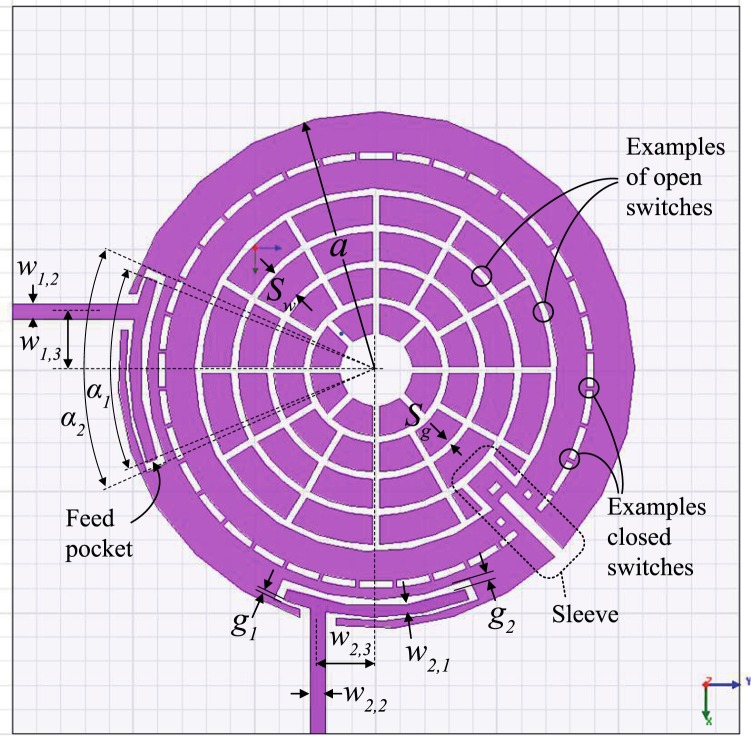
Pocket-style feeds used in an annular resonator design that applies the internal-aperture frequency-tuning concept, alongside mode-splitting. It was designed on Duroid 5880 with *ε*_*r*_ = 2.2, *a* = 20 mm, sandwiched between two substrates, each substrate with *h* ≈ 1.575 mm, and all the dimensions of the two feeds are symmetric: *a* = 21.3 mm, *w*_1,1_ = *w*_2,1_ = 0.9 mm, *w*_1,2_ = *w*_2,2_ = 1.2 mm, *α*_1_ = 44°, *α*_2_ = 48°, *w*_1,3_ = *w*_2,3_ = 4.8 mm, *g*_1_ = 0.3 mm, *g*_2_ = 0.6 mm, *s*_*w*_ = 2.4 mm (and 3.3 mm for the out-most metal loop), switch widths of 0.3 mm, and *s*_*g*_ = 0.6 mm.

**Figure 14:**
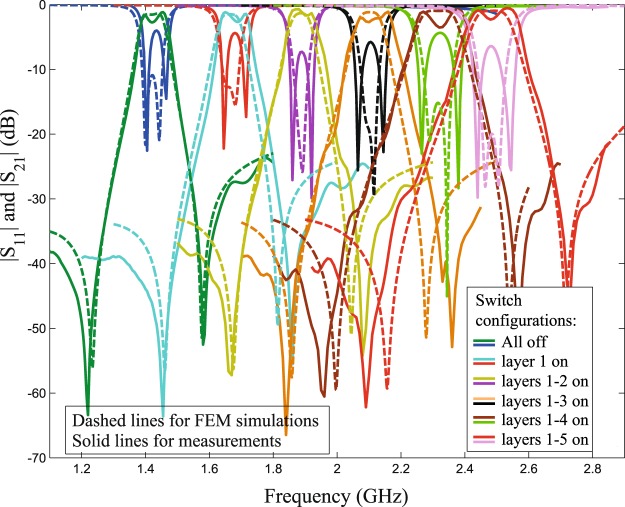
Comparison between measurements and simulations, for the pocket-style feeds resonator in Fig. 13. Note that the first layer is the outermost layer. Note that the performance (return loss and insertion loss) at the *f*_1_ and *f*_2_ frequency points has improved compared to Fig. 12 due to the buried feed configuration.

To further improve the Insertion Loss (IL) and feed impedance matching at the resonant eigenvalues (frequencies *f*_1_ and *f*_2_) in this design, the external feed coupling needs to be increased. However, edge-coupled feeds fabricated using conventional etching/milling tools would often disallow bringing the feed gap to values much smaller than 0.1 mm, forcing us to widen the span (*α*) of the feeds, to exchange more energy to/off the resonator’s edge. Even with wide spans, the structure may still not get sufficient coupling and the large feed span may cause the feed to expose more geometric changes when the resonator shape is tuning in its vicinity (in addition to causing the feed’s self-resonant frequency to appear closer to the resonator’s frequency). A more practical solution is to ‘bury’ the feeds into pockets inside the resonator’s initial periphery, which will enable more energy exchange (better external coupling) and relative geometric isolation from other features during tuning, while keeping the coupling gap feasible. Figures 11(b), 13 and 14 show a practical implementation with improved results in terms of IL and matching values (return loss) at *f*_1_ and *f*_2_ for each frequency tuning step. Note that, here, we are concerned with the matching specifically at the two split frequencies, *f*_1_ and *f*_2_, rather than the span between them. Indeed, the higher the *Q* of such resonators, the sharper the dips in |*S*_11_| (dB) will be at *f*_1_ and *f*_2_ and the shallower the curve between them. The shallowness of the curve between the two frequencies is also subject to how far the two frequencies apart.

## Analytical approximation of performance degradation

It was remarked earlier that even if the tuning elements were as ideal as conductive contacts, the performance (*Q*_0_) achieved by the resonator after geometric modification will form an upper limit on performance when any tuning elements are hosted. This important concept was reported in^4^, which also gave a theoretical model for a simplified structure that contained a single (unary) layer of switches. In this section, we discuss the analysis in more detail, including full derivations, discussion of switch distribution options, mode conversion effects and extension of derivations into multiple (*N*) layers for the perturbed circular disk case.

**Figure 15:**
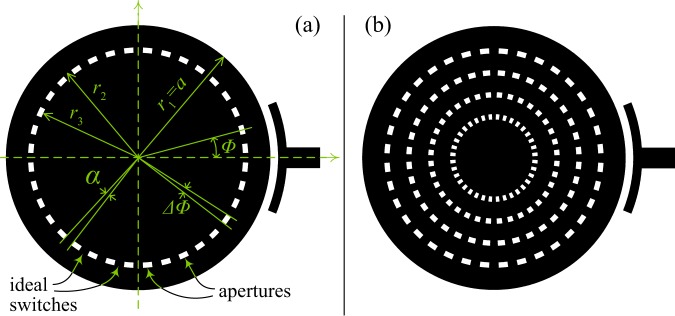
Approximate model used to estimate the effect of geometric modifications on the quality factor. In (**a**) only one (unary) layer of modification is present, whereas in (**b**) *N* = 4 layers are given as an example of higher *N* values. Radii are always denominated starting from the outer edge inwards, *r*_1_ = *a*, *r*_2_, *r*_3_, and so on. The total number of switches (or apertures) in each layer is denoted *M*.

It is clear that exact closed-form field analysis in relation to the modes resonating in structures such as those discussed above is not possible, as it entails solving the Helmholtz equation under mixed boundary conditions that are mostly inseparable and nonconforming with the coordinates. However, to gauge the degradation in performance analytically, we may use approximate perturbation methods. When we apply geometric modifications to the resonator’s structure, preparing it to host tuning elements, we effect cuts and apertures in the conductive face of the resonator. Such modifications cause losses to the dominant mode (${{\rm{TM}}}_{11}^{z}$ in the disk resonator) due to local mode conversion at geometric corners and edges (to satisfy all boundary conditions). One could attempt to roughly estimate the behavior of *Q*_0_ as we make geometric tuning to the circular resonator by considering the model geometry in Fig. 15. This is based on the classical model of a cavity with magnetic side-walls, but with the added geometric modifications to the conductive plane of the resonator.

For the dominant ${{\rm{TM}}}_{11}^{z}$ mode of propagation, the field components inside the cavity are given in cylindrical coordinates by^4,5,11^

1 $${E}_{z}=C{J}_{1}[kr]\,\cos (\varphi ),$$

2 $${H}_{\varphi }=\frac{-iCk}{{k}_{0}{Z}_{0}}{J^{\prime} }_{1}[kr]\,\cos (\varphi ),$$

3 $${H}_{r}=\frac{-iC}{{k}_{0}{Z}_{0}r}{J}_{1}[kr]\,\sin (\varphi ),$$

where *J*_1_[*x*] represents Bessel function of the first kind and the first order in *x*, and *J*′_1_[*x*] is its first derivative with respect to its argument (*x*), *k* is the wavenumber, *k*_0_ is the wavenumber in free space, *Z*_0_ is the wave impedance in free space and *C* is an arbitrary amplitude constant.

The *Q*_0_ formula is given as *Q*_0_ = *ω*(*W*_*e*_ + *W*_*h*_)/*P*_*l*_, where *W*_*e*_, *W*_*h*_ are the average stored electric and magnetic energies in the cavity’s volume, *V*, respectively, and *P*_*l*_ is the total lost power^5,6,13^. Since *W*_*e*_ and *W*_*h*_ are known to be equal at resonance, *ω*_0_, *Q*_0_ reduces to

4 $${Q}_{0}=2{\omega }_{0}\frac{{W}_{e}}{{P}_{l}}=2{\omega }_{0}\frac{{W}_{e}}{{P}_{{l}_{c}}+{P}_{{l}_{d}}+{P}_{{l}_{r}}},$$

where ${P}_{{l}_{c}}$ and ${P}_{{l}_{d}}$ are the powers lost in finite-conductivity electric-walls and in imperfect dielectric material, respectively. The term ${P}_{{l}_{r}}$ donates any other forms of loss that are implementation-dependent, such as losses due to radiation through the side-walls or parasitic coupling to enclosure and between adjacent features, which may exist in practice^3,5^. Since geometric modification will be made in the interior of the resonator and are small relative to the wavelength, with the dominant mode mainly unaffected, it is reasonable to assume that the ${P}_{{l}_{r}}$ term is approximately constant in all calculations. This assumption is consolidated by the fact that, when geometric modifications are made and small apertures appear in the resonator (as in Fig. 15), the average radiated power through such apertures is approximately zero. This is easily checked from calculating the Poynting vector and average power flow though such apertures:

$$\frac{1}{2}{\oint }_{A}{\rm{R}}{\rm{e}}\,[E\times {H}^{\ast }]\hat{z}rdrd\varphi \approx \frac{1}{2}{\oint }_{A}{\rm{R}}{\rm{e}}\,[\,-\,{E}_{z}{H}_{\varphi }^{\ast }\hat{r}+{E}_{z}{H}_{r}^{\ast }\hat{\varphi }]\hat{z}rdrd\varphi =0,$$

where *A* is the area of each aperture under consideration. Thus, if any fields are formed near the apertures, they would be reactive fields, present to satisfy the boundary conditions. This observation also agrees with Bethe’s small aperture theory in general^5,14^. Hence, radiation from such apertures can be neglected compared to ${P}_{{l}_{r}}$.

The final expression for *Q*_0_ in the unperturbed circular cavity has been given previously in^5^ assuming no radiation or coupling losses $({P}_{{l}_{r}}=0)$. For practical implementations, however, we usually have losses that will contribute to ${P}_{{l}_{r}}$ and, therefore, we should retain this term. We consider three cases: (1) unperturbed, (2) perturbed with single (unary) layer, and (3) perturbed with *N* layers, as in Fig. 15. In all models, the distribution of switches (and resulting apertures) will be taken as symmetric, since this is the expected form of implementation in general and it also happens to simplify the angular integrals involved. Derivations (see supplemented Appendix) give the following results, where we denote the unperturbed case by *Q*_0_, the unary perturbed case by *Q*_1_ and the *N*-layer perturbed case by *Q*_*N*_:

5 $${Q}_{0}{|}_{{P}_{{l}_{r}}=0}=\frac{2{\omega }_{0}{W}_{e}}{{P}_{{l}_{d}}+{P}_{{l}_{c}}}=\frac{{k}_{0}h}{2{R}_{m}{Y}_{0}+{\varepsilon ^{\prime\prime} }_{r}{k}_{0}h/{\varepsilon ^{\prime} }_{r}},$$

6 $${Q}_{0}=\frac{2{\omega }_{0}{W}_{e}}{{P}_{{l}_{d}}+{P}_{{l}_{c}}+{P}_{{l}_{r}}},$$

7 $${Q}_{1}\approx 2{\omega }_{0}\frac{{W}_{e}(1-{\gamma }_{1})}{{P}_{{l}_{d}}(1-{\gamma }_{1})+{P}_{{l}_{c}}(1-\frac{{\gamma }_{1}}{2})+{P}_{{l}_{r}}},$$

8 $${Q}_{N}\approx \frac{2{\omega }_{0}{W}_{e}(1-{\sum }_{n=1}^{N}{\gamma }_{n})}{{P}_{{l}_{d}}(1-{\sum }_{n=1}^{N}{\gamma }_{n})+{P}_{{l}_{c}}(1-{\sum }_{n=1}^{N}\frac{{\gamma }_{n}}{2})+{P}_{{l}_{r}}},$$

9 $${\rm{with}}\,{W}_{e}={|C|}^{2}\frac{\pi {\varepsilon }_{0}{\varepsilon ^{\prime} }_{r}h}{4}\frac{{a}^{2}}{2}\{(1-\frac{1}{{k}^{2}{a}^{2}}){J}_{1}^{2}[ka]\},$$

10 $${P}_{{l}_{d}}=\frac{2{\omega }_{0}{\varepsilon ^{\prime\prime} }_{r}}{{\varepsilon ^{\prime} }_{r}}{W}_{e},$$

11 $${P}_{{l}_{c}}={|C|}^{2}\frac{{R}_{m}\pi }{{k}_{0}^{2}{Z}_{0}^{2}}{k}^{2}\frac{{a}^{2}}{2}(1-\frac{1}{{k}^{2}{a}^{2}}){J}_{1}^{2}[ka],$$

where *h* is the height of the substrate, *ε*_0_ is permittivity of free-space, ${\varepsilon ^{\prime} }_{r}$ and ${\varepsilon ^{\prime\prime} }_{r}$ are the real and imaginary parts of the substrate’s complex relative permittivity, *ε*_*r*_ = ${\varepsilon ^{\prime} }_{r}-i{\varepsilon ^{\prime\prime} }_{r}$, *R*_*m*_ is the conductor’s surface resistance, *Y*_0_ = 1/*Z*_0_, with *γ*_1_ (for the unary case, *N* = 1) given by

12 $${\gamma }_{1}=\frac{M{\rm{\Delta }}\varphi }{2\pi }\frac{{[\frac{{r}^{2}}{2}\{{J^{\prime} }_{1}^{2}[kr]+(1-\frac{1}{{k}^{2}{r}^{2}}){J}_{1}^{2}[kr]\}]}_{{r}_{3}}^{{r}_{2}}}{\frac{{a}^{2}}{2}(1-\frac{1}{{k}^{2}{a}^{2}}){J}_{1}^{2}[ka]},$$

and *γ*_*n*_ (for multi-layer case, *N* > 1) given by

13 $${\gamma }_{n}=\frac{M{\rm{\Delta }}\varphi }{2\pi }\frac{{[\frac{{r}^{2}}{2}\{{J^{\prime} }_{1}^{2}[kr]+(1-\frac{1}{{k}^{2}{r}^{2}}){J}_{1}^{2}[kr]\}]}_{{r}_{2n+1}}^{{r}_{2n}}}{\frac{{a}^{2}}{2}(1-\frac{1}{{k}^{2}{a}^{2}}){J}_{1}^{2}[ka]}.$$

These simplified models do not take into account some of the complex effects (e.g. aperture couplings, curvature, fringing, air-mode/radiation changes, conversion to higher modes), but are, nonetheless, still helpful in quickly revealing the characteristic performance degradation trends for the structure under discussion.

We now evaluate the *Q*_0_ and *Q*_1_ (unary layer) performances using the derived approximations for a canonical example similar to the one given in^5^. The unperturbed resonant frequency is taken to be 4 GHz, using copper-plated Alumina substrate of ${\varepsilon ^{\prime} }_{r}$ = 9.7, $\varepsilon ^{\prime\prime}$ = 0.0002 and substrate height *h* = 1 mm. This would give ${Q}_{0}{|}_{{P}_{{l}_{r}}=0}\approx 940$. In one stripline setup, where the substrate has a radius equal to the resonator’s patch and the GND planes (all equal to *a* ≈ 7.05 mm), with magnetic side-walls, the FEM simulations (using HFSS, Ansys), extra losses $({P}_{{l}_{r}})$ caused the actual *Q*_0_ to be circa 635 (which implies ${P}_{{l}_{r}}\approx 1.025\times {10}^{-11}$ W for this example). This can, clearly, be a different value in a different implementation setup. Another way of expressing this^3,15^ is by including the extra loss terms in a quality factor of its own, and then add its effect to a *Q*_0_ that does not include any ${P}_{{l}_{r}}$ effects $({Q}_{0}{|}_{{P}_{{l}_{r}}=0})$; i.e., $1/{Q}_{0}=1/{Q}_{0}{|}_{{P}_{{l}_{r}}=0}+1/{Q}_{r}$. Both ways of expressing the extra losses effect are equivalent.

**Figure 16:**
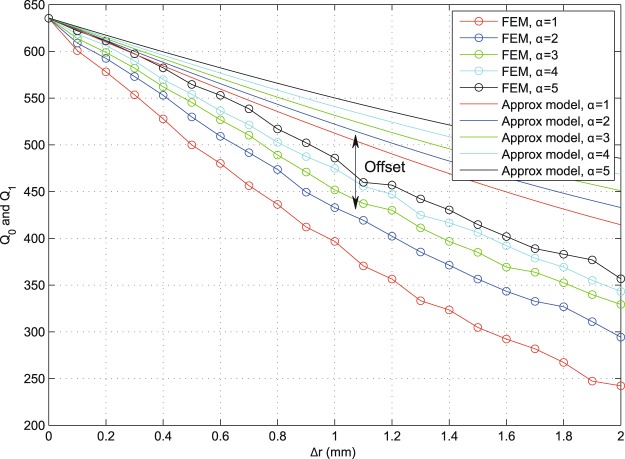
An example demonstrating frequency degradation trends for the indicative values of *N* = 1, *M* = 24, *r*_2_ = 6.1 mm and *α* ∈ [1, 5], using results obtained from numerical FEM and from the presented approximate models.

**Figure 17:**
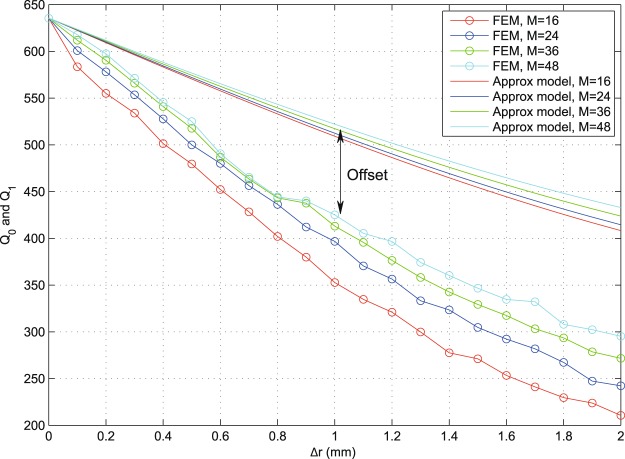
An example demonstrating frequency degradation trends for the indicative values of *N* = 1, *α* = 1°, *r*_2_ = 6.1 mm and *M* ∈ {16, 24, 36, 48}, using results obtained from numerical FEM and from the presented approximate models.

Figures 16 and 17 show examples of the degradation in performance as we tune Δ*r* = *r*_2_ − *r*_3_ and *α* (switch angular width). The general trend is more acute degradation with larger/more apertures (higher *M*, smaller *α*) and wider gaps (larger Δ*r*). Note that useful practical dimensions for the miniature switches (e.g., RF MEMS switches^16,17^) should be much smaller than the wavelength and with, say, Δ*r* < 1 mm and *α* < 10°, for the given example. Typical values are chosen here as *α* ∈ [1°, 5°] and Δ*r* ∈ [0, 2] mm.

Throughout the results, it is clear that the approximate models show similar characteristic performance trends as those found numerically (FEM method), but with an offset that can be seen in the presented curves. In addition to being attributed to the simplifying approximations made in our modelling, such as the absence of fringing and coupling effects, this offset is mainly due to the approximate calculations that only take into account the dominant mode and do not include energy lost to higher modes via mode conversion on geometric features. This is an expected price for keeping the model relatively simple and analytic. The validity of the used HFSS models was confirmed earlier^15^, where the initial smooth resonator shapes (unperturbed) were observed to operate on fundamental modes with fringing effects (largely free of mode-conversion effects). The measured fringing agreed with standard models in the literature^15^. As we add local geometric alteration in such geometries to host the tuning elements, the only key difference is the added local discontinuities, which are known to generate mode conversion losses^2–7^. Such losses are difficult to measure individually/directly due to the excitation of multiple higher (evanescent) modes at different local spots within the resonator’s body^2–7^.

**Figure 18:**
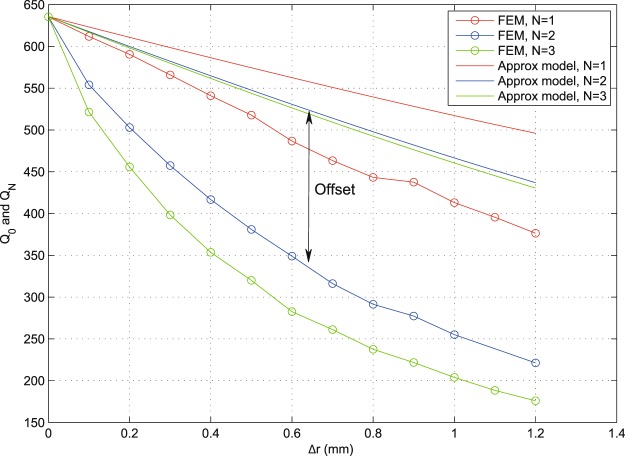
Results obtained from numerical FEM and from the approximate models, for the indicative values of *M* = 36, *α* = 1°, *r*_2_ = 6.1 mm, *r*_4_ = 4.1 mm, *r*_6_ = 2.1 mm and *N* ∈ [1, 3] (multi-layer). Despite the offset due to the discussed simplified model assumptions, characteristic trends are preserved; note the correlation in relative differences between the *N* = 1, 2 curves and the *N* = 2, 3 curves in both sets of results.

Figure 18 shows an indicative example of *α* = 1 and *M* = 36 switches (apertures) with *N* ∈ [1, 3] layers. We can now see the characteristic cumulative effect of multiple layers, as expected (*Q*_*N*_ degrades with *N*). It is noted that the *Q*_*N*_ approximation based on the simplified assumptions above exhibits larger offsets compared to numerical FEM results than it did for the unary layer case (*N* = 1), even though the characteristic trends are still observable. For example, it is noted how the performances of *N* = 2 and *N* = 3 are closer to each other than those for *N* = 1 and *N* = 2 in both FEM and model results. This indicates an interesting behavior: most of modal distortion happens due to the first layer (*r*_2_, *r*_3_), which presents a fundamental change in geometry compared to the original shape. Such performance trends highlight the importance of optimizing the geometric strategies used to host the tuning elements, even before such elements are inserted and their additional losses incorporated. An example of the losses later added by the tuning elements, on top of the loss due to geometric effects, is given in^4^. By noticing the key geometric parameters in the equations for *Q*_0_, *Q*_1_ and *Q*_*N*_, and performance figures generated therefrom, such as Figs 16–18, a resonator designer will be able to understand the trade-offs at play and choose the optimal geometric parameters that will approach the sought specifications. Even though the discussion above has used the circular case to demonstrate the analysis, similar analyses and conclusions may be drawn for other structure shapes.

## Conclusion

Geometric perturbations that enable eigenmode manipulation in dual-mode resonators are important because they allow us to tune resonant systems that are found in many applications in physical sciences and engineering. The geometric choices made to implement such tuning, however, can degrade a resonator’s performance significantly. Novel geometric techniques were discussed in this paper, highlighting important aspects that link performance degradation to geometry. The performance degradation inherent in such structures was also discussed and predicted using approximate analytical models. Key advantages of the presented novel structures include electronic geometric tunability for frequency splitting and shifting, as well as the use of buried feeds to improve insertion loss and return loss performance.

## Supplementary information

Appendix

## References

[1] Naji, A. & Soliman, M. Center frequency stabilization in planar dual-mode resonators during mode-splitting control. *Sci. Rep.* **7** (2017). 10.1038/srep43855 PMC5341017 28272422

[2] Cameron, R., Mansour, R. & Kudsia, C. *Microwave Filters for Communication Systems* (Wiley Blackwell, 2018).

[3] Hong, J.-S. & Lancaster, M. *Microstrip Filters for RF/Microwave Applications* (Wiley Blackwell, 2001).

[4] Naji A, Warr P, Beach M, Morris K (2011). A fundamental limit on the performance of geometrically-tuned planar resonators. IEEE Trans. Microw. Theory Techn..

[5] Collin, R. *Foundations for Microwave Engineering* (Wiley Blackwell, 2000).

[6] Pozar, D. *Microwave Engineering* (John Wiley and Sons, 2004).

[7] Okoshi, T. *Planar Circuits for Microwaves and Lightwaves* (Springer-Verlag, 1985).

[8] Wolff I (1972). Microstrip bandpass filter using degenerate modes of a microstrip ring resonator. Electron. Lett..

[9] Lugo C, Papapolymerou J (2005). Bandpass filter design using a microstrip triangular loop resonator with dual-mode operation. IEEE Microw. Wirel. Compon. Lett..

[10] Athukorala L, Budimir D (2009). Compact Dual-Mode Open Loop Microstrip Resonators and Filters. IEEE Microw. Wirel. Compon. Lett..

[11] Watkins J (1969). Circular resonant structures in microstrip. Electron. Lett..

[12] www.ansoft.com.

[13] Ginzton, E. *Microwave Measurements* (McGraw-Hill, 1957).

[14] Bethe H (1944). Theory of diffraction by small holes. Phys. Rev..

[15] Naji A, Warr P (2012). Independence of the unloaded Q of a planar electromagnetic resonator from its shape.IEEE Trans. Microw. Theory Techn..

[16] Santos, H. *RF MEMS Circuit Design for Wireless Communications* (Artech House, 2002).

[17] Rebeiz, G. *RF MEMS: Theory, Design and Technology* (Wiley Blackwell, 2003).

