# Publisher Correction: Geometrical Perturbation Techniques and Approximate Analysis for Eigenmode Splitting and Shifting in Electromagnetic Planar Dual-Mode Resonators

**DOI:** 10.1038/s41598-019-49268-w

**Published:** 2019-09-10

**Authors:** Adham Naji, Paul A. Warr

**Affiliations:** 1 Electrical and Electronic Engineering Department, University of Sheffield, Sheffield, S10 2TN UK; 2 Communication Systems and Networks (CSN), Faculty of Engineering, University of Bristol, Bristol, BS8 1UB UK

Correction to: *Scientific Reports* 10.1038/s41598-018-37787-x, published online 20 February 2019

This Article contains errors in two of the Figures. In Figure 2, the shapes in the first row (a-d) are incorrectly all shown as the same shape. In Figure 8, there is a pixel-shift distortion of the graphics. The correct Figures 2 and 8 appear below as Figures 1 and 2 respectively.

**Figure 1:**
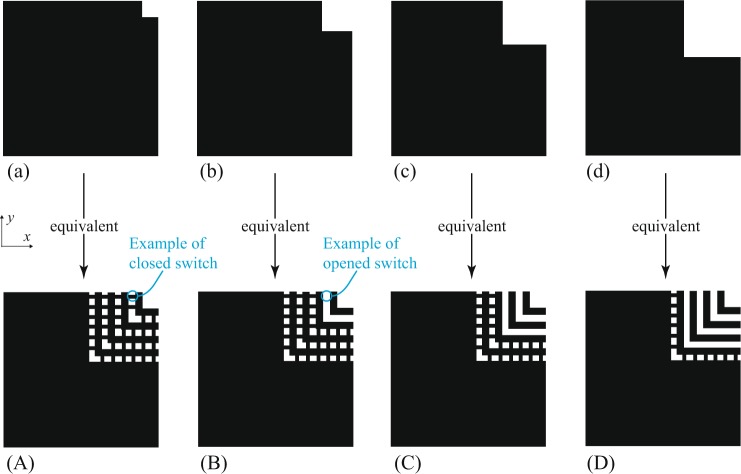
An example of a basic geometric method to achieve stepped corner perturbation inside a dual-mode square resonator. Feeds are assumed to be aligned with the x and y axes, but are not shown. The idealized switches here are small conductive strips that are switched on/off by being present/absent. As the perturbation gets larger, the eigenmode splitting is increased. Cases (**a–d**) are equivalent to configurations (**A–D**).

**Figure 2:**
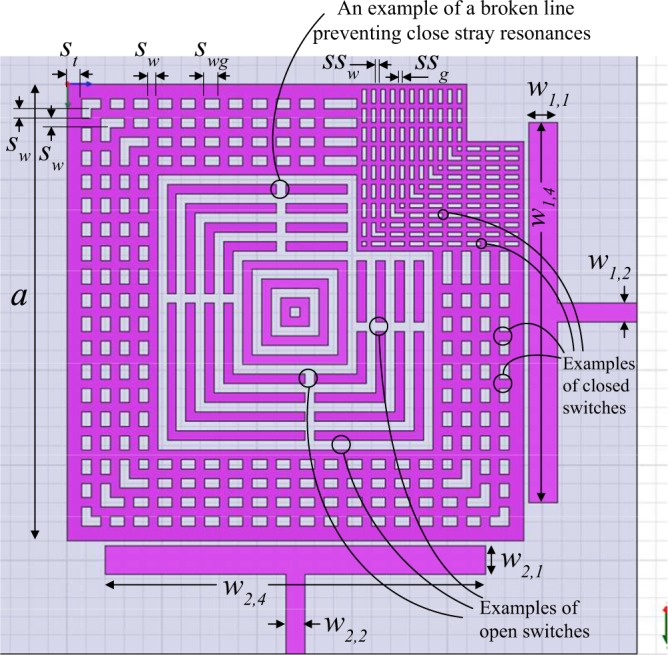
A square resonator design that applies the internal-aperture frequency-tuning concept, alongside mode-splitting.

