# Supplementary material for: Geometrical Perturbation Techniques and Approximate Analysis for Eigenmode Splitting and Shifting in Electromagnetic Planar Dual-Mode Resonators
Source: Sci Rep. 2019 Feb 20;9:2417. doi: 10.1038/s41598-018-37787-x (PMC6382774; doi:10.1038/s41598-018-37787-x)
Supplement: Supplementary file 1 — Appendix [file 41598_2018_37787_MOESM1_ESM.pdf]

Appendix for

Geometrical Perturbation Technique and Approximate Analysis for Eigenmode Splitting and Shifting in Electromagnetic Planar Dual-Mode Resonators

Adham Naji and Paul Warr

(Note: equation numbering is consistent with the main article)

Appendix

For the unperturbed case, the average stored electric energy is given by $W_e = \frac{\varepsilon_0 \varepsilon_r'}{4} \int\limits_V E \cdot E^* dV.$ (14)

Substituting equations (1–3) in (14) gives

$$W_e = |C|^2 \frac{\varepsilon_0 \varepsilon_r'}{4} \int\limits_0^h \int\limits_0^{2\pi} \int\limits_0^a J_1^2[kr] \cos^2(\phi) r dr d\phi dz \tag{15}$$

$$= |C|^2 \frac{\pi \varepsilon_0 \varepsilon_r' h}{4} \frac{a^2}{2} \left\{ J_1'^2[ka] + \left(1 - \frac{1}{k^2 a^2}\right) J_1^2[ka] \right\} \tag{16}$$

$$= |C|^2 \frac{\pi \varepsilon_0 \varepsilon_r' h}{4} \frac{a^2}{2} \left\{ \left(1 - \frac{1}{k^2 a^2}\right) J_1^2[ka] \right\}, \tag{17}$$

Equation (16) is found from equation (15) using the known Bessel integral relation

$$\int x J_n^2[ax] dx = \frac{x^2}{2} \left\{ J_n'^2[ax] + \left(1 - \frac{n^2}{a^2 x^2}\right) J_n^2[ax] \right\}. \tag{18}$$

Equation (16) is reduced to (17) by the application of the $J_1'^2[kr] = 0$ Neumann boundary condition at the edge of the resonator, where $r = a$ (i.e., at the magnetic-wall).

P_{l_d} is the power lost in the imperfect dielectric, which is

$$P_{l_d} = \frac{\omega_0 \varepsilon_r'' \varepsilon_0}{2} \int\limits_V E \cdot E^* dV = \frac{2\omega \varepsilon_r''}{\varepsilon_r'} W_e. \tag{19}$$

The power lost in the conductor is the integral of the ohmic loss on the surface of the metal of the resonator and its GND plane. Thus, if R_m is the surface resistance of the metal, and top and bottom boundaries have identical areas, P_{l_c} is given by

$$P_{l_c} = 2 \times \frac{R_m}{2} \int\limits_0^{2\pi} \int\limits_0^a |H_r|^2 + |H_\phi|^2 r dr d\phi \tag{20}$$

$$= \frac{|C|^2 R_m}{k_0^2 Z_0^2} (\pi) \int\limits_0^a \left\{ k^2 J_1'^2[kr] + \frac{J_1^2[kr]}{r_2} \right\} r dr. \tag{21}$$

After some manipulation, and by making use of equation (18), equation (21) reduces to

$$P_{l_c} = |C|^2 \frac{R_m \pi}{k_0^2 Z_0^2} \int\limits_0^a k^2 r J_1^2[kr] dr \tag{22}$$

$$= |C|^2 \frac{R_m \pi}{k_0^2 Z_0^2} k^2 \frac{a^2}{2} \left(1 - \frac{1}{k^2 a^2}\right) J_1^2[ka]. \tag{23}$$

If P_{l_r} was negligibly small ($P_{l_r} \to 0$), then the expression for Q_0 can be reduced to

$$Q_0|_{P_{l_r}=0} = \frac{2\omega_0 W_e}{P_{l_d} + P_{l_c}} = \frac{k_0 h}{2 R_m Y_0 + \varepsilon_r'' k_0 h / \varepsilon_r'}, \tag{24}$$

which is the same result obtained in reference [5] in main article. However, for practical implementations, we retain the P_{l_r} term to reflect any implementation-dependent losses (see discussion in the analytical approximation section in the main article) that may be experienced in practice. Thus, a more practical expression for Q_0 is given by

$$Q_0 = \frac{2\omega_0 W_e}{P_{l_d} + P_{l_c} + P_{l_r}}, \tag{25}$$

where P_{l_r} is estimated from practical measurements or approximate numerical simulations, and W_e, P_{l_d} and P_{l_c} are found from equations (17), (19) and (23).

Now, Q_1 for the unary (N=1) layer of geometric modification [Figure 15(a)] can be approximately calculated after removing the aperture volumes and surfaces from the integral domains in energy and power calculations, as follows

$$Q_1 \approx 2\omega_0 \frac{\breve{W}_e}{\breve{P}_{l_c} + \breve{P}_{l_d} + \breve{P}_{l_r}}, \tag{26}$$

where $\breve{W}_e = W_e - \Delta W_e$, $\breve{P}_{l_d} = P_{l_d} - \Delta P_{l_d}$, $\breve{P}_{l_c} = P_{l_c} - \Delta P_{l_c}$ and $\breve{P}_{l_r} \approx P_{l_r}$. Thus,

$$Q_1 \approx 2\omega_0 \frac{(W_e - \Delta W_e)}{(P_{l_d} - \Delta P_{l_d}) + (P_{l_c} - \Delta P_{l_c}) + P_{l_r}}, \tag{27}$$

where, for each aperture (i), the changes in stored energy and lost powers are (note that $\Delta P_{l_d} = 2\omega_0 \varepsilon_r'' \Delta W_e / \varepsilon_r'$):

$$\Delta W_e = |C|^2 \frac{\varepsilon_0 \varepsilon_r'}{4} \int_0^h \int_{\phi_i - \frac{\Delta\phi}{2}}^{\phi_i + \frac{\Delta\phi}{2}} \int_{r_3}^{r_2} J_1^2[kr] \cos^2(\phi) r dr d\phi dz \tag{28}$$

$$\Delta P_{l_c} = \frac{R_m}{2} \int_{\phi_i - \frac{\Delta\phi}{2}}^{\phi_i + \frac{\Delta\phi}{2}} \int_{r_3}^{r_2} |J_r|^2 + |J_\phi|^2 r dr d\phi. \tag{29}$$

Note that this conductor loss takes into account the difference the aperture caused in the patch face only (the ground plane is unaffected).

For all the apertures in the layer, we perform the integration, with each aperture (i) centered at ϕ_i, and add the results. However, for symmetric deployment of the M apertures angularly around the center, with each having an equal angular width of $\Delta\phi_i = \Delta\phi$, the integration of the angular parts of the integrands, over all apertures, becomes

$$\sum_{i=1}^{i=M} \int_{\phi_i - \frac{\Delta\phi}{2}}^{\phi_i + \frac{\Delta\phi}{2}} \left\{ \begin{array}{c} \cos^2\phi \\ \sin^2\phi \end{array} \right\} d\phi = \frac{1}{2} \left[M\Delta\phi \mp \sin\Delta\phi_i \sum_{i=1}^{i=M} \cos 2\phi_i \right] = \frac{1}{2} M\Delta\phi \tag{30}$$

Using these results in equations (28) and (29) gives

$$\Delta W_e = \gamma_1 W_e, \quad \Delta P_{l_d} = \gamma_1 P_{l_d}, \quad \Delta P_{l_c} = \frac{\gamma_1}{2} P_{l_c}, \tag{31}$$

where the proportionality factor γ_1 is given by

$$\gamma_1 = \underbrace{\frac{M\Delta\phi}{2\pi}}_{\text{angular ratio (linear)}} \underbrace{\frac{\left[\frac{r^2}{2} \left\{ J_1'^2[kr] + \left(1 - \frac{1}{k^2 r^2}\right) J_1^2[kr] \right\} \right]_{r_3}^{r_2}}{\frac{a^2}{2} \left(1 - \frac{1}{k^2 a^2}\right) J_1^2[ka]}}_{\text{radial ratio (nonlinear)}} \tag{32}$$

$$\Rightarrow Q_1 \approx 2\omega_0 \frac{W_e (1 - \gamma_1)}{P_{l_d}(1 - \gamma_1) + P_{l_c}(1 - \frac{\gamma_1}{2}) + P_{l_r}} \tag{33}$$

Note that $\gamma_1 \in [0, 1[$ and represents the ratio of the apertures' effective modal area to the total modal area of the original patch (unperturbed). Equation (33) is used in the above sections as equation (7).

For the general case of N layer modification, we need to include the effects of all apertures, were we now have a γ_n factor ($n = 1, 2, 3, \cdots$) for each of the N layers, and with the nth layer limited by the radii r_{2n} and r_{2n+1}. Thus, the first layer will have $n = 1$ and radii pair (r_2, r_3), the second layer will have have $n = 2$ and radii pair (r_4, r_5), and so on. Note that $r_1 = a$ by definition. This gives

$$Q_N \approx 2\omega_0 \frac{W_e (1 - \sum_{n=1}^{N} \gamma_n)}{P_{l_d}(1 - \sum_{n=1}^{N} \gamma_n) + P_{l_c}(1 - \sum_{n=1}^{N} \frac{\gamma_n}{2}) + P_{l_r}}, \tag{34}$$

where, for each layer, γ_n is given by

$$\gamma_n = \frac{M\Delta\phi}{2\pi} \frac{\left[\frac{r^2}{2} \left\{ J_1'^2[kr] + \left(1 - \frac{1}{k^2 r^2}\right) J_1^2[kr] \right\} \right]_{r_{2n+1}}^{r_{2n}}}{\frac{a^2}{2} \left(1 - \frac{1}{k^2 a^2}\right) J_1^2[ka]} \tag{35}$$

Equation (34) is used in the analytical approximation section as equation (8).
